# Molecular diet studies of water mites reveal prey biodiversity

**DOI:** 10.1371/journal.pone.0254598

**Published:** 2021-07-29

**Authors:** Adrian A. Vasquez, Obadeh Mohiddin, Zeyu Li, Brittany L. Bonnici, Katherine Gurdziel, Jeffrey L. Ram

**Affiliations:** 1 Healthy Urban Waters, Department of Civil and Environmental Engineering, Wayne State University, Detroit, Michigan, United States of America; 2 Cooperative Institute for Great Lakes Research, School for Environment and Sustainability, University of Michigan, Ann Arbor, Michigan, United States of America; 3 Department of Physiology, Wayne State University School of Medicine, Detroit, Michigan, United States of America; 4 Genome Sciences Core, Wayne State University, Detroit, Michigan, United States of America; Universidade Federal de Lavras, BRAZIL

## Abstract

Water mites are diverse aquatic invertebrates that provide potentially important ecosystem and economic services as bioindicators and mosquito biocontrol; however, little is known about water mite digestive physiology, including their diet in nature. Water mites, much like their spider relatives, liquefy their prey upon consumption. This results in the absence of morphologically identifiable prey in water mite mid-gut. Previous studies have reported associations in the field of water mites with presumed prey and laboratory observations of water mites feeding on specific organisms offered for ingestion; however, the present work aims to determine what water mites have ingested in nature based on molecular studies of gut contents from freshly collected organisms from the field. To elucidate water mite prey, we used next-generation sequencing to detect diverse cytochrome oxidase I DNA barcode sequences of putative prey in the guts of 54 specimens comprising two species of *Lebertia* and a few specimens of *Arrenurus* (2) and *Limnesia* (1). To our knowledge this is the first molecular study of the diets of water mites as they feed in nature. While the presence of chironomid DNA confirmed previous observations of midge larvae as part of the diets of *Lebertia*, we also found the DNA of diverse organisms in all four species of water mites, including the DNA of mosquitoes in 6 specimens of *Lebertia* and a large number of previously unknown prey, especially from oligochaete worms. These studies thereby reveal a greater diversity of prey and a potentially broader significance than previously appreciated for water mites in aquatic food webs. Molecular studies like this can detect water mite predators of mosquito larvae and add knowledge of water mite predatory contributions to freshwater food webs.

## Introduction

Water mites are small carnivorous arachnids that are fully adapted as adults to water. They have a complex life cycle in which the larvae of most water mite species parasitize adult aquatic insects, such as mosquitoes, as the insect adult emerges from its aquatic pupal stage. Water mites are one of the most diverse aquatic invertebrates, with over 10,000 described species; however, relatively little is known about water mites, especially in North America [1]. It is estimated that up to half of the species have not even been described, named [1] or barcoded [2]. A similar lack of knowledge exists about the identity and diversity of the microscopic or small macroscopic prey that water mites consume including oligochaetes which are reported here [3].

In contrast to other aquatic organisms such as fish, water mites have largely been ignored in food web depictions of aquatic ecosystems as a result of scientific neglect for freshwater invertebrates when compared to freshwater vertebrates [4]. For example, the current Great Lakes food web descriptions from the National Oceanic and Atmospheric Association (NOAA) are incomplete and do not include many important benthic predators such as water mites (https://www.glerl.noaa.gov/res/projects/food_web/food_web.html). Water mites are considered “underappreciated” aquatic predators, and this work aims to raise the profile of water mite research, especially in North America [5].

Even the analysis of water mite diets has largely been limited in the past to testing what water mites will ingest in the laboratory [5–7]. Acarologists have previously listed a variety of water mite prey based on field observations and laboratory feeding experiments. While we can easily show that various species of water mites in the laboratory will attack and consume larvae of chironomids, mosquitoes, and ostracods, that is far from revealing what they ingest in nature. Water mites are presumed to prey on a range of invertebrates available in the habitats they reside in. The list includes copepods, cladocerans, chironomids, mosquito larvae and others [5, 6, 8]. For example, *Lebertia* has been reported to feed on Diptera larvae [8]. Some studies have suggested that mite predation are so great that they compete with fish for food [9] and may limit the spread of invasive species due to their parasitism [10]. Water mites are also parasitic on adult aquatic insects including mosquitoes, dragonflies, chironomids and beetles but this is restricted to the larval stage [11]. While some work has revealed observational records of water mite adults feeding on mosquito larvae in small temporary ponds [12] the present study is the first comprehensive investigation of the diet diversity of water mites, and the only study to our knowledge that has used high throughput next-generation sequencing (NGS) for this purpose in water mites.

Water mites are members of Arachnida, the large group of invertebrates that includes spiders, ticks and terrestrial mites. They all share a similar way of feeding, by external digestion that results in a liquefied meal. It is impossible to identify prey items by microscopy after liquefication; therefore, this study applies a molecular method as the best means to identify the water mite prey, by sequencing the partially digested DNA of ingested prey. Previous work on spiders’ diets from coastal habitats used a combination of molecular analysis of diet DNA and carbon isotopes to differentiate whether their prey was coming from a terrestrial or marine source [13]. In our study we chose to use the molecular approach since adult water mites are fully aquatic, microscopic, and difficult to observe directly in their natural habitat.

NGS has become a standard way to study diet contents in other animals groups, especially when gut contents are too fragmented to study morphologically [14]. NGS has been used to study diets in fish, spiders, ticks, and parasitic wasps [13, 15–17]. Martin et al. [18] sequenced DNA extracted from the gut of *Hygrobates* water mites that had previously been eating a single species of Chironomid in laboratory-based-feeding experiments. Their experiments showed that *Hygrobates* mites, offered chironomid prey in a laboratory setting, fed on the chironomids, and subsequent PCR amplification with chironomid-specific 18S primers detected chironomid DNA signals for at least 24 hours after feeding [18]. This study hypothesizes that using NGS technology on DNA from freshly collected water mites would detect the DNA of more diverse ingested species beyond that shown in laboratory-based feeding experiments and thereby reveal the natural constituents of water mite diets. Two species of *Lebertia* were chosen as the focus of this molecular water mite diet studies because of their year-round seasonal availability, previous reports on their predation of aquatic Diptera larvae of ecological importance, abundance and diversity in a local freshwater lagoon [7, 19–22].

## Methods

### Ethics statement

JLR is Director of the Belle Isle Aquarium research field lab and had a collecting permit from the Michigan Department of Natural Resources for Belle Isle. The sample collections did not involve any endangered or protected species.

### Water mite collection and DNA extraction

Water mites were collected on multiple dates between July 2016 and March 2017 (see S1 Table) from Blue Heron Lagoon (latitude 42.344661, longitude -82.959324), on Belle Isle, an island park of the State of Michigan located in the Detroit River, by dragging a 200 μm net along the bottom and through emergent vegetation in depths of 0.5–1 m, washed on a 250 μm sieve, and transported to the Wayne State University Belle Isle Aquarium Field Research Laboratory within 30 minutes of collection. Live water mites were immediately picked on sorting trays and preserved by blanching followed by immersion in 70–90% ethanol and storage at 4 ^o^C, as previously described [20]. Mites were sorted according to genus and species (*Lebertia* was our study model) and isolated from the rest of the sample as done in our prior studies [20]. Each mite was washed with ethanol to avoid cross-contamination from other organisms. Mites that were damaged or pierced during sorting were not selected for this analysis. Whole mite DNA was extracted by puncturing individual water mites with sterile minutien pins to allow water mite lysate, including gut contents, to ooze out. DNA was obtained from the punctured mites by lysing and extracting with Proteinase K solution overnight (8–12 hours at 57 ^o^C), and purifying DNA using a Qiagen DNA spin-column protocol (https://www.qiagen.com/us/resources/resourcedetail?id=6b09dfb8-6319-464d-996c-79e8c7045a50&lang=en), followed by storage of the extracted DNA at -20 ^o^C [20]. A voucher of the exoskeleton was retained for morphological analysis and is being held at our laboratory in the Physiology Department, Wayne State University School of Medicine. DNA extraction methods using the Qiagen DNA spin-column protocol were applied to various chironomids and mosquitoes without puncturing the organism. Their sequences were generated when needed for comparative purposes and positive controls.

### Molecular gut analysis of laboratory-fed water mites

To validate our approach, DNA was extracted from *Lebertia* mites after feeding them insect larvae. Water mites (*Lebertia*) were placed in 6-well plates (Fisher Scientific, IL) and provided chironomid and mosquito larvae. Early instar fruit fly (*Drosophila*) larvae were also offered as prey to water mites by submerging the larvae in the well with the mite. Photos were taken of mites feeding on or attempting to feed on larvae.

After mites had fed on and released the prey item the mites were blanched and DNA was extracted as described above. DNA was then amplified with the mitochondrial cytochrome oxidase I (COI) primer pair mLep and LCO1490 (top of Table 1), previously used as “non-arachnid arthropod-specific COI primers.” These primers generate a product of 332 bp [13, 23, 24]. The more broad “metazoan” DNA barcoding primers; COI forward and reverse HCO2198 and LCO1490 primers generate a product of 658 bp in comparison [25]. Prey DNA was also extracted and amplified with the same primers. Control experiments amplified DNA from the legs of mites, which excludes gut tissue. Amplicons were sequenced by Sanger sequencing (GeneWiz, Plainfield, NJ).

**Table 1:** Primers.

Name of Primers	Primer Sequence	Annealing Temperature	Reference
mLep LCO1490	5’-CCTGTTCCAGCTCCATTTTC-3’ 5’-GGTCAACAAATCATAAAGATATTGG-3’	50 ^○^C	[23]
mLep+TAG LCO1490+TAG	5’-TACGGTAGCAGAGACTTGGTCTCCT GTTCCAGCTCCATTTTC3’ 5’ACACTGACGACATGGTTCTACA GGTCAACAAATCATAAAGATATTGG-3’	50 ^○^C	

### Next-generation sequencing

For next-generation sequencing, DNA extracted from 54 freshly collected water mites, comprising 21 *Lebertia quinquemaculosa*, 30 *L*. *davidcooki*, 1 *Limnesia*, and 2 *Arrenurus* specimens, were amplified with the versions of the mLep and LCO1490 primers tagged with Fluidigm CS1 and CS2 Illumina adapters fused to their 5’ ends, as listed in Table 1 (bottom). *Limnesia* and *Arrenurus* were two outgroup water mites chosen to compare with *Lebertia* but were not the focus of this study. The amplicons were sent to the Michigan State University RTSF Genomics Core for next-generation sequencing on an Illumina MiSeq V2 platform. Specimen-specific index sequences were ligated onto all PCR products, which were purified and sequenced and the resultant quality-filtered fastq files analyzed, as follows: Amplicons were processed to remove dNTPs, primer dimers, and other small side-products (less than 100 bp in size), using the Agencourt AMPure XP system (Beckman). PCR with sample-indexed primers targeting the CS1/CS2 oligos was performed to add dual-indexed, Illumina compatible adapters at the ends of the PCR products. The sample-indexed PCR products were batch-normalized using Invitrogen SequalPrep DNA normalization plates and the recovered products pooled. The pool was quality-controlled and quantified using a combination of Qubit dsDNA HS, Caliper LabChipGX HS DNA and Kapa Illumina Library Quantification qPCR assays. It was loaded on an Illumina MiSeq v2 standard flow cell and sequenced in a 2x250 bp paired-end format using a v2 500 cycle reagent cartridge. Primers complementary to the Fluidigm CS1/CS2 oligonucleotides were added to appropriate wells of the reagent cartridge to act as primers for the forward, reverse and index sequencing reads. Base calling was done by Illumina Real Time Analysis (RTA) v1.18.54, and output of RTA was demultiplexed and converted to FastQ format with Illumina Bcl2fastq v2.19.0.

### Bioinformatics and statistics

FastQ files were demultiplexed using Perl software. Because the left and right paired-end reads covered more than the whole sequence (i.e., the left and right paired reads overlap with one another), the left and right end reads were merged using FLASH (https://ccb.jhu.edu/software/FLASH/) [26]. The cutoff for a successful merge was set as minimal overlap,10 bp; maximum overlap, 150 bp, minimal mismatch ratio, 0.25; and only those pairs that were successfully merged were kept for further analysis. Then, assembled sequences were sorted into operational taxonomic unit (OTU) clusters using CD-HIT (http://www.bioinformatics.org/cd-hit/) [27], with the sequence similarity parameter set to >97% identity to the representative sequence of each OTU cluster. Further analysis was conducted only on clusters represented by at least 4 sequences. Each OTU was BLASTed (ver 2.6.0+) against the GenBank database (date = 3 June, 2018) to identify the closest identity in that database, using self-written Perl scripts to identify the top 5 hits in GenBank and only the hits with query coverage >50% and identity >80% were kept. In some cases where the top 5 hits were only at family level, BLAST analysis was repeated manually to determine if a specific genus or species could be identified within one or two percent identity of the top hit. Bioinformatics assistance and advice was provided by Wayne State University’s Applied Genomics Technology Center (https://genomesciencescore.wayne.edu/). MEGA6 [28] was used to compare various sequences, including alignments, pairwise comparisons, and construction of neighbor-joining trees. CD-HIT clusters of various sizes were sorted and graphed in pie charts on Excel^©^ spreadsheets, which were also used for histogram analysis. Mann-Whitney non-parametric statistical test calculations used an on-line calculator (https://www.socscistatistics.com/tests/mannwhitney/default2.aspx). Mann-Kendall and Yuen’s test calculations were performed by S. Sawilowsky (College of Education, Wayne State University, using R and Minitab^®^ 18, respectively).

### Code availability

The code used in this study was deposited in GitHub at https://github.com/GeoCoderL/mite-diet.

### Reference sequences and sequence data availability

The high throughput nucleotide sequence data was uploaded to GenBank as Accession IDs MW605229—MW615071 administered by the National Center for Biotechnology Information (https://submit.ncbi.nlm.nih.gov/subs/sra/) and tohttps://digitalcommons.wayne.edu/physio_frp/2. Genus and species names, their authorities and years, and their family and subfamily identities were verified by reference to https://www.catalogueoflife.org/ and through the use of the Web of Science reference list (https://clarivate.com/webofsciencegroup/solutions/web-of-science/) and NCBI taxonomy browser (https://www.ncbi.nlm.nih.gov/Taxonomy/Browser/wwwtax.cgi).

## Results

### *Lebertia* water mite species from Blue Heron Lagoon, Detroit

**Fig 1:**
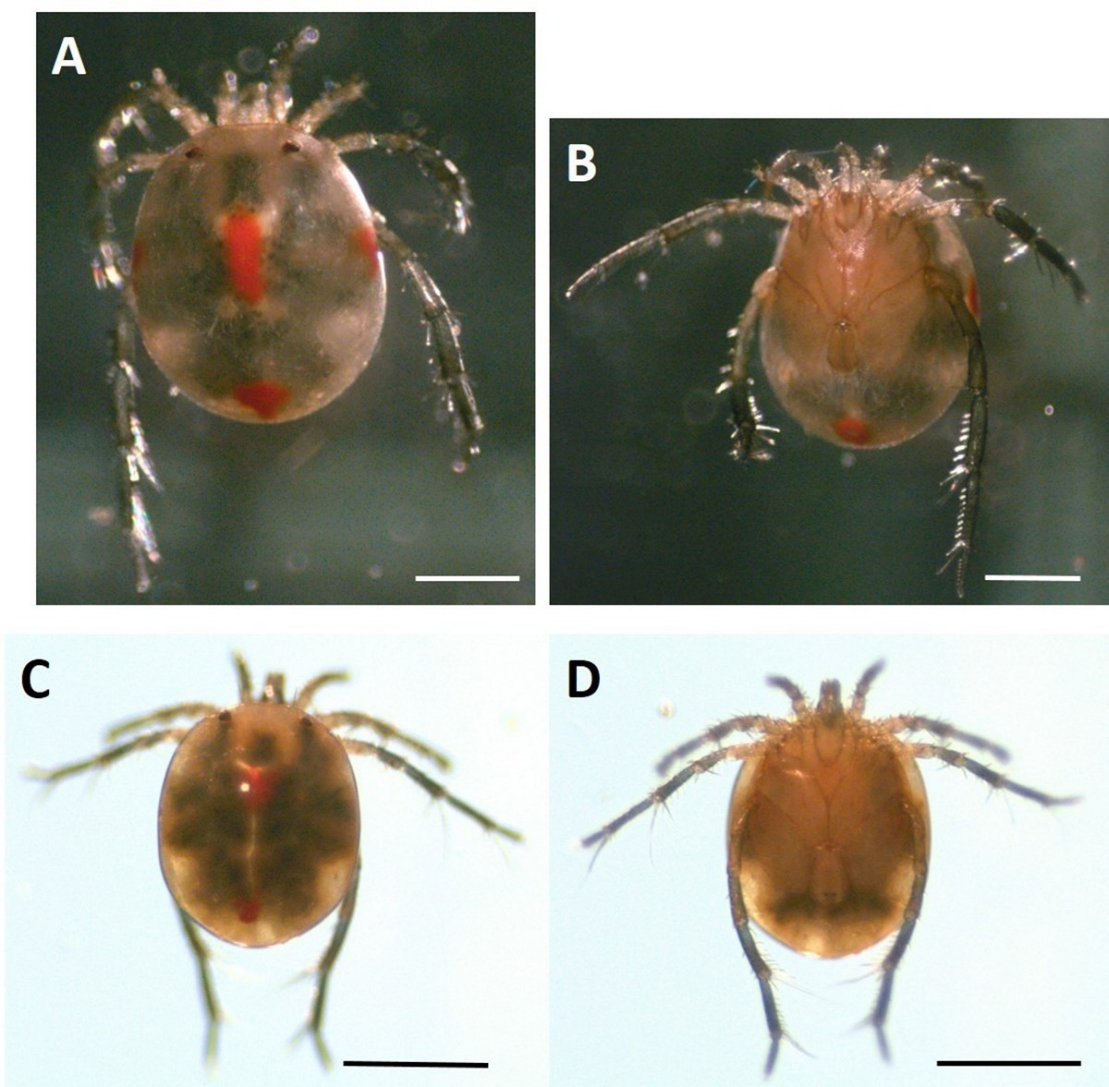
Two species of *Lebertia* water mites found in Blue Heron Lagoon. A, *L*. *quinquemaculosa* dorsal and B, ventral. C, *L*. *davidcooki* dorsal and D, ventral. Calibration bars = 500 μm.

Blue Heron Lagoon is home to at least two species of *Lebertia* water mites which we have reported in previous studies [20] and further confirmed in this work by DNA barcoding using the “universal metazoan” barcode primers (HCO2198 and LCO1490) of Folmer et al. [25, 29]. We identified the barcodes of *L*. *quinquemaculosa* and *L*. *davidcooki* (Fig 1A, 1B, and 1C, 1D, respectively; GenBank accession IDs MG773261.1 and MG773262.1, respectively). *L*. *quinquemaculosa* and *L*. *davidcooki* COI barcodes differed from one another by a pairwise difference of 16%. *L*. *davidcooki* differs from all previous *Lebertia* barcodes by > 11% and is close in morphology to *L*. *inaequalis* [30], whose barcode (HQ919893.1) differs from *L*. *davidcooki* by 13%.

### Laboratory-fed *Lebertia* and preliminary DNA analysis of freshly caught specimens

DNA extracted from mites that had recently ingested chironomid or mosquito prey in the laboratory reliably yielded DNA sequences that matched the prey (Table 2). In control experiments, DNA extracted from legs of *Lebertia* (therefore, not including the gut region) amplified as expected with HCO2198 and LCO1490 primers, yielding the expected water mite sequences. Leg DNA amplified by mLep/LCO1490 primers yielded no PCR product, as expected. Tests of mLep/LCO1490 primers with fish DNA were also negative.

**Table 2:** Prey DNA in mites after laboratory feeding.

Predator	Prey	GenBank match*:
*Lebertia quinquemaculosa*	*Culex pipiens*	*C*. *pipiens* Q100%; ID99%
*L*. *quinquemaculosa*	*C*. *pipiens*	*C*. *pipiens* Q100%; ID99%
*L*. *quinquemaculosa*	*C*. *pipiens*	*C*. *pipiens* Q98%; ID99%
*Lebertia davidcooki*	Chironomid	*Chironomidae* sp. Q100%; ID97%
*Lebertia davidcooki*	Chironomid	*Cricotopus* sp.Q99%; ID97%

*GenBank Query coverage (Q) and percent identity (ID) of mLep/LCO1490 PCR products

**Fig 2:**
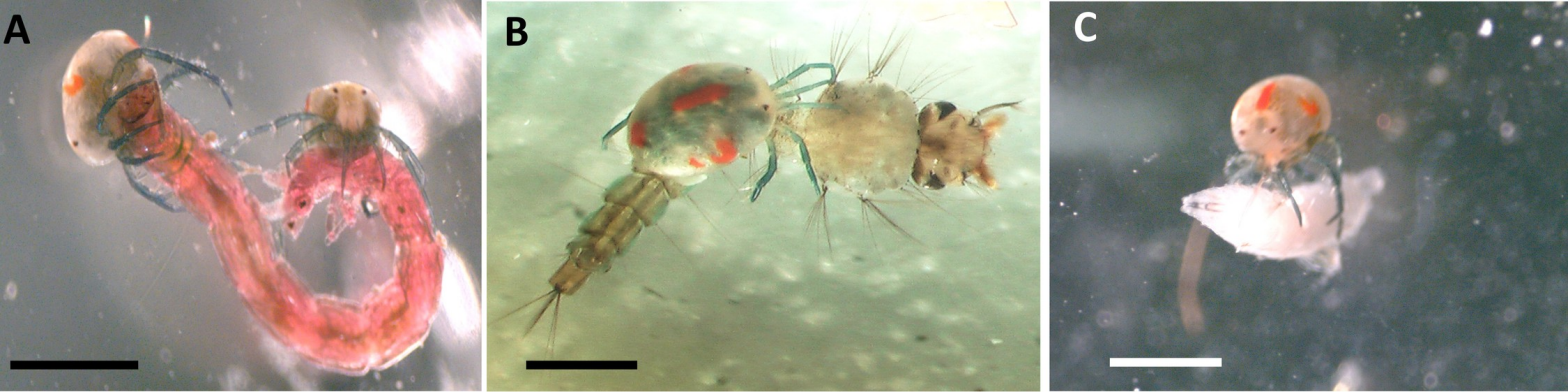
*Lebertia quinquemaculosa* preying upon organisms in laboratory feeding experiments. A, *Chironomus riparius*; B, *Culex pipiens*, and C, *Drosophila melanogaster*. Calibration bars = 1 mm.

Laboratory feeding experiments with *L*. *quinquemaculosa* (Fig 2A) demonstrate that *L*. *quinquemaculosa* will attack and ingest dipteran aquatic larvae, such as chironomid (midge fly, Fig 2A) and mosquito larvae (Fig 2B), consistent with the only prey listed for Lebertioidea mites in an authoritative review [8]. When offered larvae of *Drosophila*, an animal that they would never expect to encounter in the aquatic environment, they also attacked it (Fig 2C). To determine what water mites eat in nature, a preferred method would be to observe them directly attacking organisms in their natural environment (difficult given their microscopic size) or, as we have done, examining what animals freshly collected from the environment have ingested.

DNA extracted from six freshly caught *Lebertia* with primers mLep/LCO1490, designed to amplify non-mite DNA, showed that field-collected specimens contained diverse non-mite sequences (S2 Table). For example, non-mite DNA from *Lebertia* 6-BHL022317 had 100% identity and 93% query coverage to *Paratanytarsus* sp. (Chironomidae) and non-mite DNA from *Lebertia* 2-BHL022317 had 99% identity though only 75% query coverage to *Paratanytarsus* sp. Unexpectedly, non-mite DNA from the other three *Lebertia* had closest matches to oligochaetes (*Slavina* and *Nais*, respectively, but with only 88% sequence match) and another non-insect (e.g., *Diaphanosoma*, albeit with only 42% query coverage), which have not previously been associated with *Lebertia* diets. The sequence chromatograms were often complex, possibly reflecting overlapping sequences from multiple prey organisms. Therefore, we next used Illumina high-throughput sequencing to identify multiple individual prey DNA sequences in freshly collected *Lebertia*.

### Next-generation sequencing of non-mite DNA in *Lebertia* and other water mites

**Fig 3:**
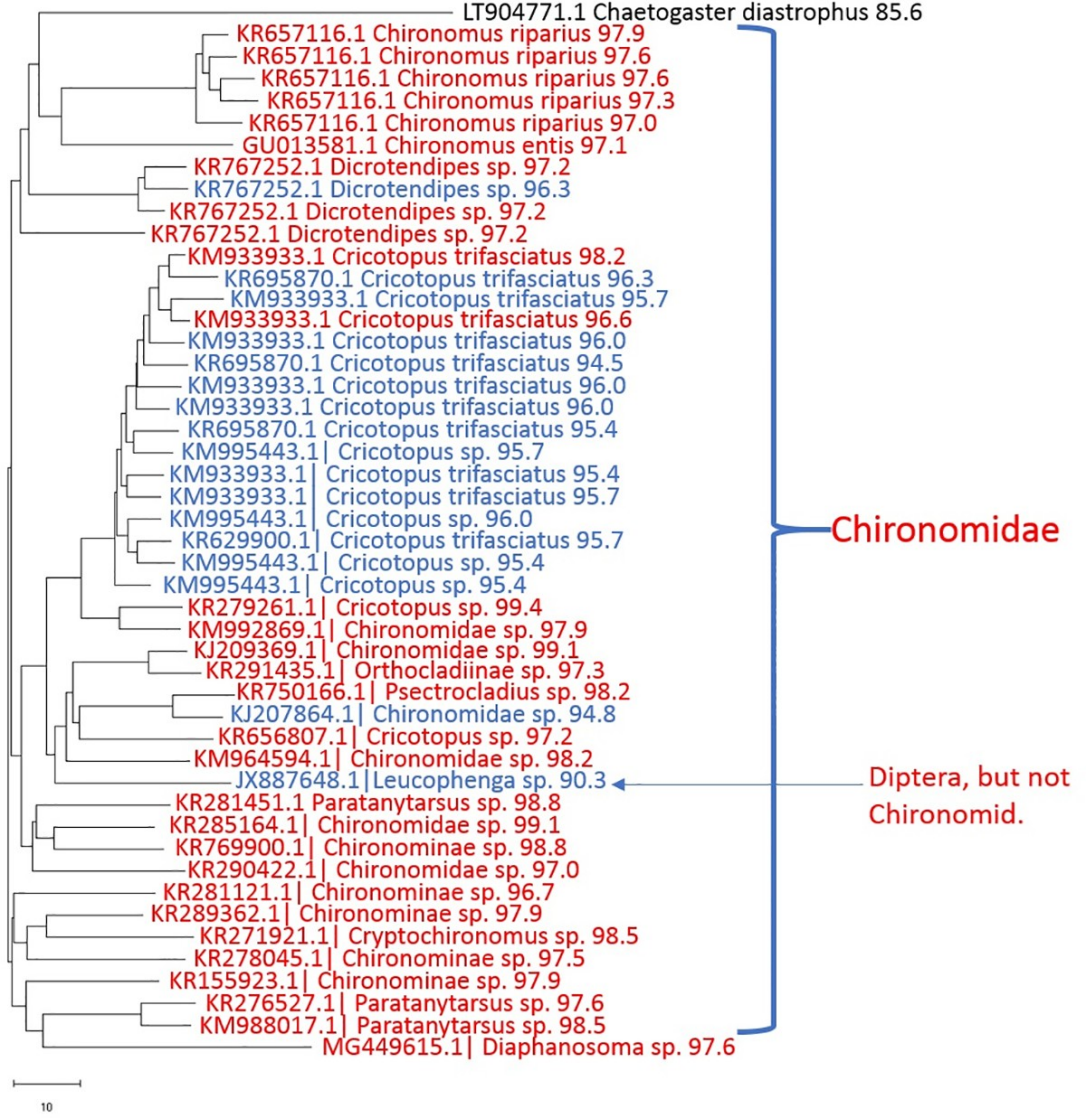
Neighbor-joining tree of non-mite DNA OTUs in representative *Lebertia davidcooki* mite of PCR products amplified with mLep/LCOI primers-Illumina adapter primers. Each branch is labeled with the “best hit” accession ID in GenBank, taxon name, and the percent identity of the OTU to its best hit. Mostly chironomids in a specimen of L. davidcooki. Color code: red, identities >96.5%; blue, identities 90% - 96.5%; black, identities 80% - 90%. All OTUs represent >4 sequences.

**Fig 4:**
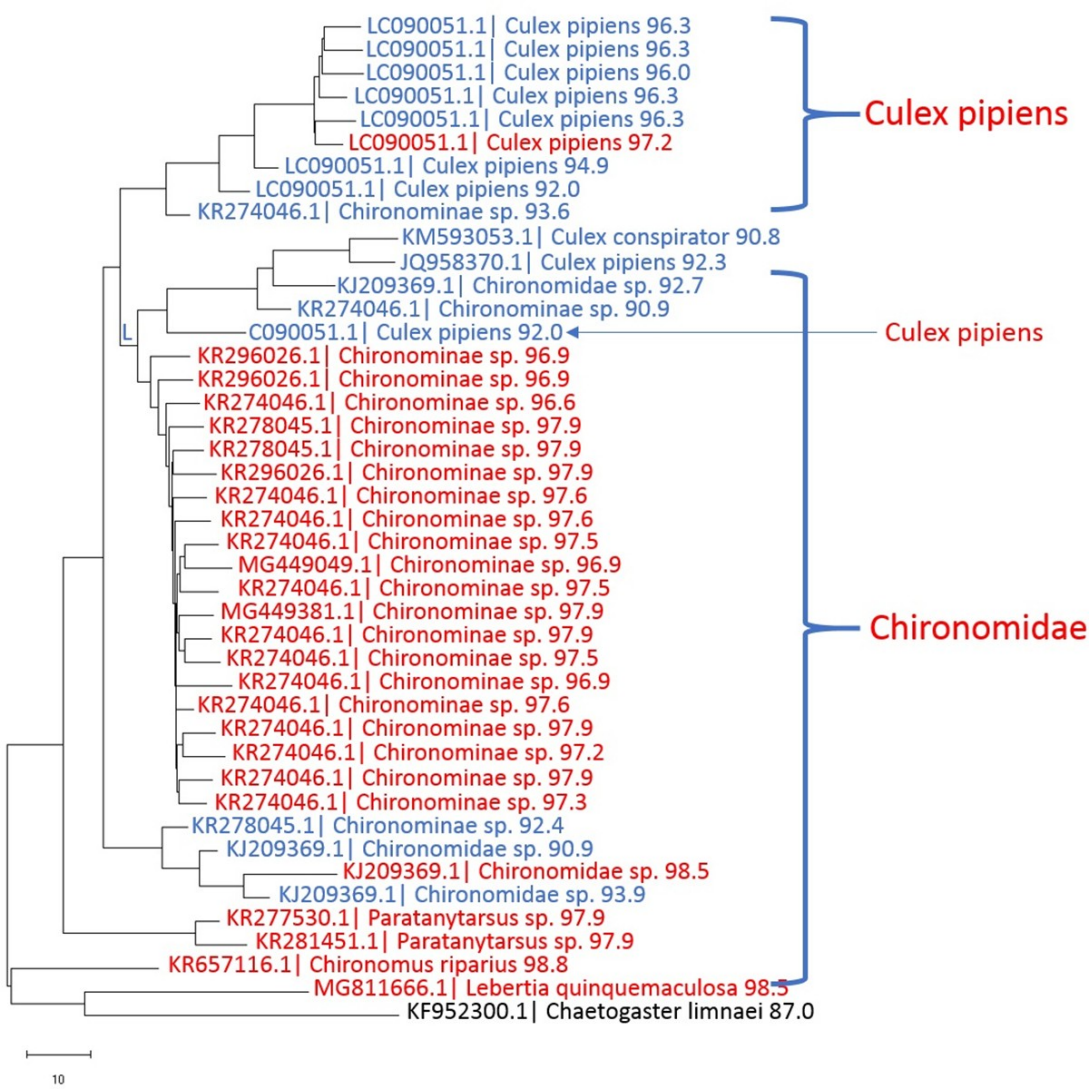
Neighbor-joining tree of non-mite DNA OTUs in representative *Lebertia quinquemaculosa* mite of PCR products amplified with mLep/LCOI primers-Illumina adapter primers. Each branch is labeled with the “best hit” accession ID in GenBank, taxon name, and the percent identity of the OTU to its best hit. Mostly chironomid and mosquito DNA in a specimen of *L*. *quinquemaculosa*. Color code: red, identities >96.5%; blue, identities 90% - 96.5%; black, identities 80% - 90%. All OTUs represent >10 sequences.

**Fig 5:**
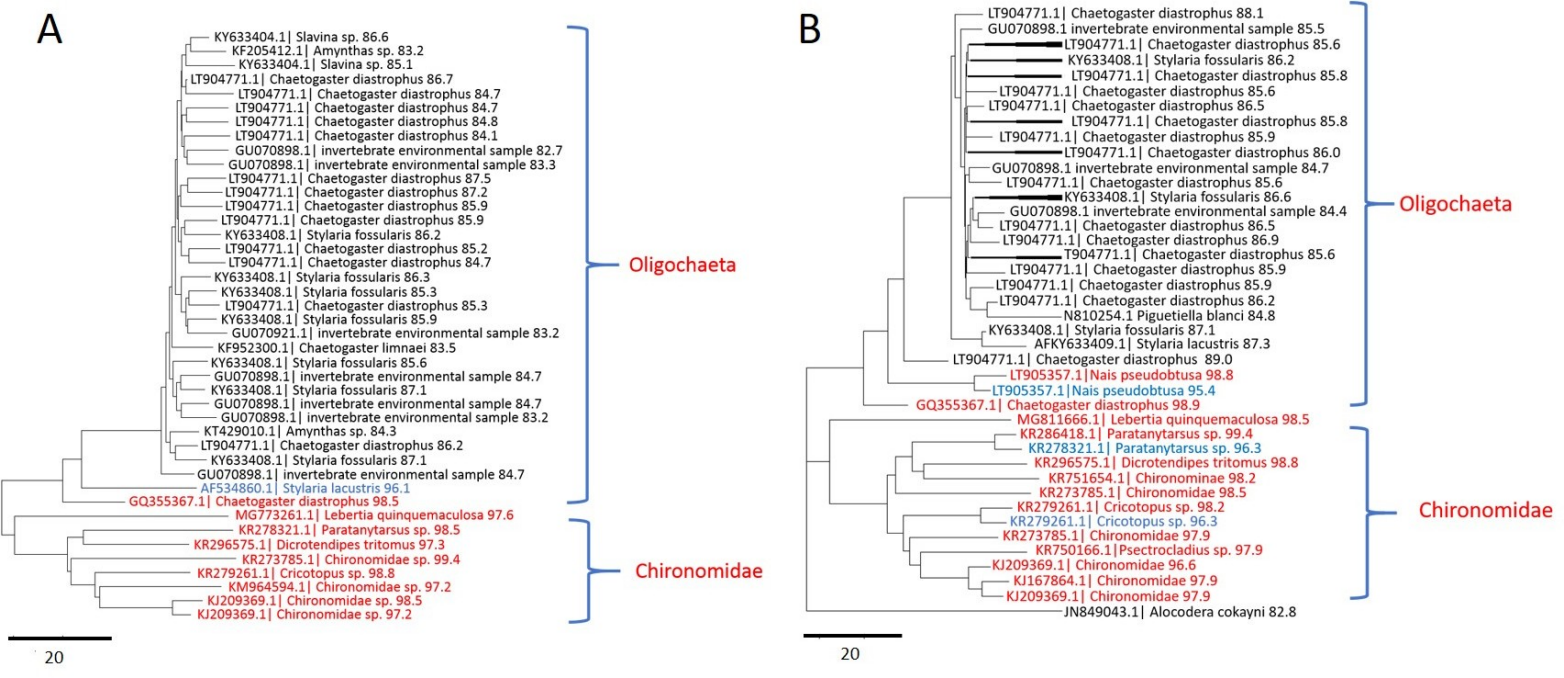
Neighbor-joining tree of non-mite DNA OTUs in representative *Lebertia quinquemaculosa* mites of PCR products amplified with mLep/LCOI primers-Illumina adapter primers. Each branch is labeled with the “best hit” accession ID in GenBank, taxon name, and the percent identity of the OTU to its best hit. A & B, A mix of oligochaetes and chironomids in specimens of *L*. *quinquemaculosa*. Color code: red, identities >96.5%; blue, identities 90% - 96.5%; black, identities 80% - 90%. All OTUs represent >4 sequences in A and >10 sequences in B.

The next-generation sequencing results show a more diverse diet of *Lebertia* than any previous study. Neighbor-joining trees of sequences from representative water mites show specimens in which OTUs were predominantly chironomids (Fig 3 from *L*. *davidcooki* and Fig 4, from *L*. *quinquemaculosa*) and others with a mix of chironomids and oligochaetes (Fig 5 from *L*. *quinquemaculosa*).

### Chironomid DNA in *Lebertia*

The diet profile of a representative *Lebertia davidcooki* water mite that was feeding primarily on chironomids is shown in Fig 3. Most of the 45 OTUs that matched chironomid sequences in Fig 3 had sequence identities to species in GenBank that were greater than 97%, including identities of 97.9%, 97.2%, 98.2%, 98.2%, 98.8%, and 98.5% obtained for *Chironomus riparius*, *Dicrotendipes* sp., *Cricotopus trifasciatus*, *Psectrocladius* sp., *Paratanytarsus* sp., and *Cryptochironomus* sp., respectively. In addition to the 45 OTUs that matched chironomids, this *L*. *davidcooki* specimen also had one oligochaete, one cladocera and one non-chironomid dipteran OTU. The diet profile of a *Lebertia quinquemaculosa* specimen in Fig 4 also contains many Chironomids but most identifications beyond 97% were only up to the subfamily level of Chironominae. In addition to 30 chironomid OTUs, this specimen also included 10 *Culex pipiens*, 1 *Culex conspirator*, 1 oligochaete and 1 *Lebertia* OTU. The percent identities for the many different Chironomid genera found in all specimens of *L*. *davidcooki and L*. *quinquemaculosa* are summarized in Table 3.

**Table 3:** Best reference genera matches to chironomid DNA found in the mite genus *Lebertia*.

Chironomid genus	*L*. quinquemaculosa* (out of 20 specimens)	*L*. davidcooki* (out of 29 specimens)
***Chironomus***	**SG**	**SGF**
***Cricotopus***	**SG**	**SG**
***Cryptochironomus***	**S**	**SG**
***Dicrotendipes***	**SG**	**SG**
***Orthocladius***		**SG**
***Paratanytarsus***	**SG**	**SG**
***Psectrocladius***	**S**	**SG**
***Tanytarsus***	**S**	
***Coelotanypus***		**S**
***Glyptotendipes***	**G**	**GF**
***Kiefferulus***	**F**	**F**
***Parachironomus***		**SG**
***Paracladopelma***		**GF**
***Phaenopsectra***		**S**
***Rheotanytarsus***		**S**
***Rietha***	** F**	

*Shown only for sequence clusters (OTUs) accounting for >0.1% of non-mite DNA sequences. Key to abbreviations: S, >96.5% identity; G, 90% to 96.5% identity; F, 80 to 90% identity to “best hit” GenBank reference sequences.

### Oligochaete DNA in *Lebertia*

Although Chironomid larvae were already known to be part of the diet of *Lebertia*, these studies show that some animals also had a large amount of oligochaete DNA. *S*ome oligochaete OTUs had sequence identities >90% (examples are *Chaetogaster diastrophus* 98.5% in Fig 5A; *Nais pseudobtusa*, 98.8% in Fig 5B; and *Amphichaeta raptisae* 91% - 94% in data from seven *L*. *davidcooki*. and three *L*. *quinquemaculosa*). However, most sequences whose closest matches in GenBank were oligochaetes often had sequence identities below 90% compared to previously barcoded oligochaetes (summarized in Table 4). These include *Slavina* sp., *Chaetogaster* spp., *Stylaria fossularis*, and others seen in Fig 5. The sequences of most *Chaetogaster* and *Stylaria*, and all *Amynthas*, *Piguetiella*, and *Slavina* matched reference sequences no better than 80 to 90% (Table 4).

**Table 4:** Best reference gene matches to oligochaete DNA found in the mite genus *Lebertia*.

Oligochaete genus	*L*. quinquemaculosa* (out of 20 specimens)	*L*. davidcooki* (out of 29 specimens)
***Amphichaeta***	**G**	**GF**
***Chaetogaster***	**SGF**	**F**
***Stylaria***	**F**	**F**
***Nais***	**SG**	**G**
***Piguetiella***	**F**	
***Slavina***	**F**	
***Amynthas***	**F**	

*Shown only for sequence clusters accounting for >0.1% of non-mite DNA sequences. Key to abbreviations: S, >96.5% identity; G, 90% to 96. 5% identity; F, 80 to 90% identity to “best hit” GenBank reference sequences.

### DNA of mosquitoes and other taxa

Mosquito (*Culex pipiens*) sequences, when present (e.g., Fig 4), usually had species level matches with >97% identity. The diet profile of the *Lebertia quinquemaculosa* specimen shown in Fig 4 indicates that although Chironominae made up most of the diet constituents of this animal, it had also fed on *Culex* spp., as represented by 10 sequences identified as *Culex pipiens* and 1 sequence identified as *Culex conspirator*. Altogether, 6 mite specimens (2 *L*. *davidcooki* and 4 *L*. *quinquemaculosa*) exhibited *Culex* spp sequences.

**Fig 6:**
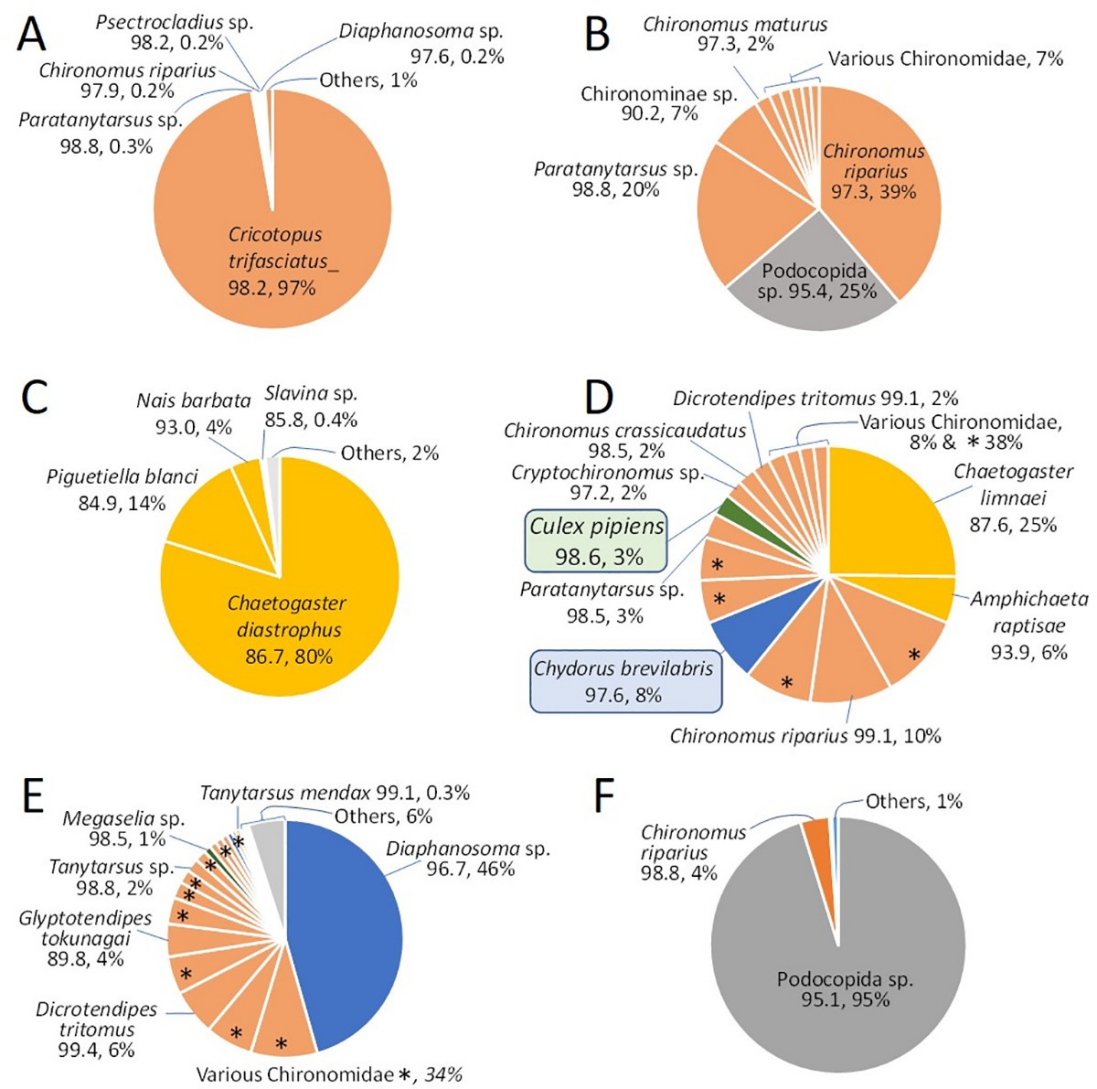
Abundance of OTUs within representative water mites. (A, B) *L*. *davidcooki*; (C-E) *L*. *quinquemaculosa*; (F) *Arrenurus*. Pie sections are proportional to the percent abundance in that mite and labeled with the name of the “best hit” reference sequence, percent identity of the OTU with the reference sequence, and it’s percent abundance. Pie sections with * are OTUs with best matches to a variety of Chironomidae or identified only to that level as the best hit.

Less common still among these specimens are sequences from other taxa, including ostracods, amoeba and the cladoceran *Diaphanasoma* sp., (97.6% identity) which was present in the *Lebertia davidcooki* in Fig 3, whilst the cladoceran *Chydorus brevilabris* was observed in *Lebertia quinquemaculosa* in Fig 6D. Fig 5B includes the amoeba *Alocodera*, 87.9% identity. Fig 6B includes *Podocopida* sp. (95% identity), which was present in seven specimens of *L*. *davidcooki* and both specimens of *Arrenurus*.

### Relative abundances of the various taxa

Each branch of the above trees represents an OTU that was observed in 4 or more specimens; however, a better indication of the relative abundance of specific OTUs in the mite diet is the cluster-sizes (number of sequences) of each OTU. In Fig 6, cluster sizes of OTUs with identical “best hit” reference taxon were summed and represented by each slice of the illustrated pie charts, thereby indicating the relative abundance of various taxa. Thus, while the tree in Fig 3 has many branches that match the non-biting midge *Cricotopus trifasciatus* sequence 95% to 98%, the pie chart of sequence abundances for the same specimen (Fig 6A) shows the most common sequence (97% of the sequences) is the one that has a 98% identity to the reference.

**Fig 7:**
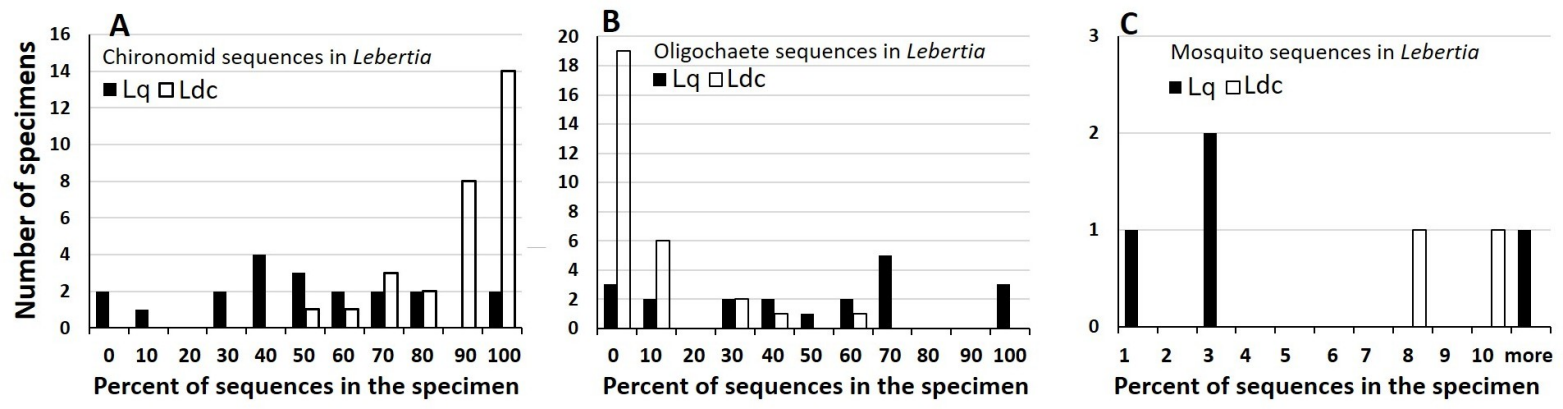
Histograms of frequency of percent abundances of sequences. (A) chironomid, (B) oligochaete, and (C) mosquito (*Culex pipiens*) OTUs in 20 *L*. *quinquemaculosa* (Lq, black bars) and 29 *L*. *davidcooki* (Ldc, white bars). The frequency of “no mosquito sequence” (bin “0”, not shown in (C)) was 16 *L*. *quinquemaculosa* and 27 *L*. *davidcooki*. Bins are 10% wide for A & B, 1% wide for C. Abundances are significantly different between *L*. *quinquemaculosa* and *L*. *davidcooki* for chironomids (p<0.00001, Mann-Whitney U test) and oligochaetes (p<0.00001, Mann-Whitney U test) but not mosquitoes (p>0.05).

The pie charts in Fig 6 illustrate diverse diets in *Lebertia*. These include specimens of the mite *L*. *quinquemaculosa* in which most non-mite sequences were from oligochaetes (Fig 6C); another with diverse sources including chironomids, worms, mosquitoes (*Culex pipiens*, 98.6% identity, 3% of the sequences), and a cladoceran (*Chydorus brevilabris*, 97.6% identity, 8% of the sequences) (Fig 6D); and another in which the cladoceran *Diaphanosoma* sp. (96.7% identity) accounted for 46% of the sequences (Fig 6E). Mosquito DNA was found in both mite species *L*. *davidcooki* (2 specimens) and *L*. *quinquemaculosa* (4 specimens, Figs 6D and 7C). For the seven specimens of *L*. *davidcooki* (e.g., Fig 6B) that had *Podocopida* sp. (with sequence identities ranging from 93 to 96%), this ostracod accounted for a mean of 16% of the sequences. Similarly, in the specimen of the mite *Arrenurus*, illustrated in Fig 6F, more than 95% of its non-mite sequences were ostracod *Podocopida* sp. (95.1% identity). Another Dipteran observed as prey of the mite *L*. *quinquemaculosa* was *Megaselia sp*. with a percent match of 98.5% identity (see Fig 6E).

### The two species of *Lebertia* differ in dietary composition and seasonal presence

The percent abundances of chironomid, oligochaete, and mosquito sequences in both *Lebertia* species are summarized in Fig 7. Chironomids, the most frequently cited prey of *Lebertia* [8], were abundant in both species, albeit in higher proportion in *L*. *davidcooki* (median = 89% of sequences) than in *L*. *quinquemaculosa* (median = 47%; Fig 5A). Conversely, oligochaete sequences (Fig 5B) were present more often in the mite *L*. *quinquemaculosa* (17 of 20 animals; median = 47% of sequences) than in *L*. *davidcooki* (10 of 29 animals; median = 0%). Even for the ten specimens of *L*. *davidcooki* with measurable oligochaete DNA the median abundance was only 5%, much lower than for *L*. *quinquemaculosa*.

**Fig 8:**
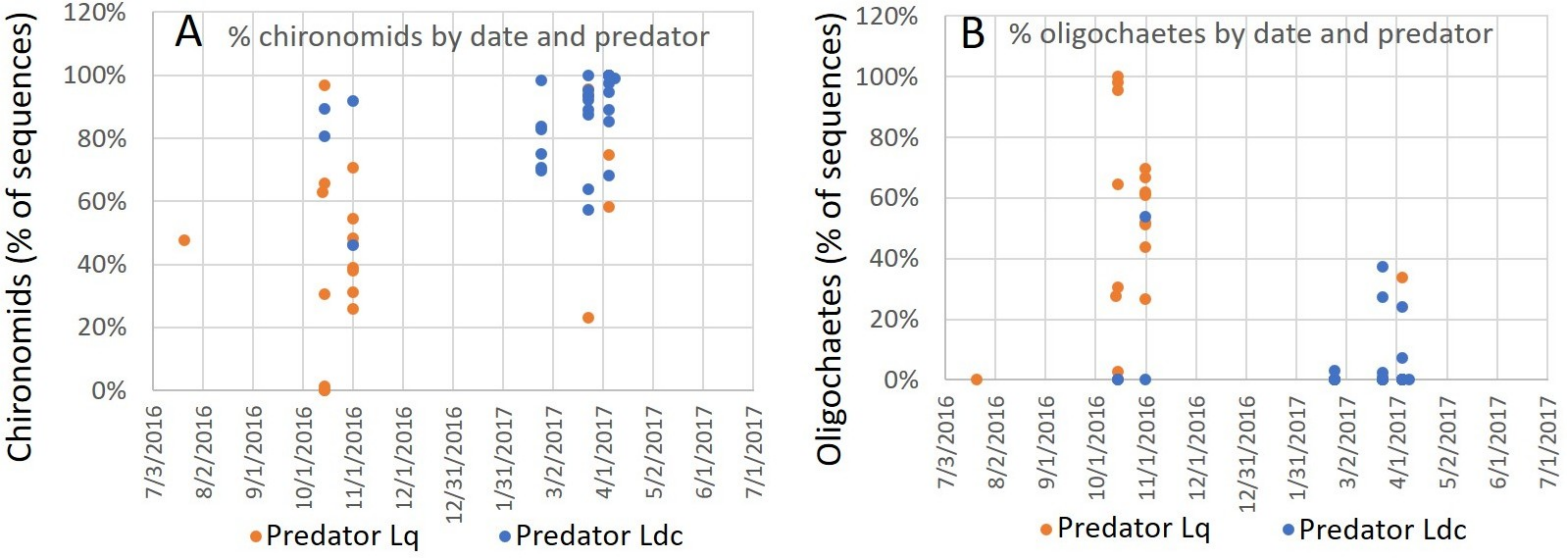
Seasonal distribution of water mite species and the percent abundances of sequences of (A) chironomid and (B) oligochaete in 20 *L*. *quinquemaculosa* (Lq, orange points) and 29 *L*. *davidcooki* (Ldc, blue points). Some points are not visible where others are superimposed on them. Differences over time analyzed by the seasonal Mann-Kendall test for trend were significant for oligochaetes in *L*. *quinquemaculosa* (p = 0.031) but not for other comparisons. In (A) *L*. *davidcooki* had a higher percentage of chironomid sequences than *L*. *quinquemaculosa* (Yuen’s test, p = 0.00001). In (B), *L*. *quinquemaculosa* had a higher percentage of oligochaete sequences than *L*. *davidcooki* (Yuen’s test, p = 0.0002).

A preliminary statistical analysis of these data using the non-parametric Mann-Whitney test indicated that the higher percentage of chironomids in the diet of *L*. *davidcooki* v. *L*. *quinquemaculosa* and oligochaetes in the diet of *L*. *quinquemaculosa* v. *L*. *davidcooki* was significant (p<0.00001 for both comparisons). However, this difference might be attributable to seasonal differences in the presence of the two species of mites and their prey. As illustrated in Fig 8, *L*. *quinquemaculosa* accounted for 90% of the *Lebertia* specimens collected in the fall of 2016 but only 15% of the *Lebertia* collected in late winter and early spring 2017 (significantly different from *L*. *davidcooki*, p<0.0001, Fisher exact test). Due to unequal variances in the distribution of prey organism sequences in their diets, a parametric test was inappropriate. Analysis of the data using Yuen’s method [31], (with Winsorized standard errors and symmetric 20% trimming, the generally accepted default), showed that the distribution of non-mite sequences between *L*. *davidcooki* and *L*. *quinquemaculosa* was significantly different for both chironomid (T_y_ = 15.34, df = 6.275, p = 0.00001) and oligochaete (T_y_ = 12.02, df = 5.18, p = 0.0002) sequences. Application of the seasonal Mann-Kendall test [32] for trend determined that the higher percentage of oligochaetes in the diet of *L*. *quinquemaculosa* in the fall was significant (p = 0.031) while seasonal trends in the diet of *L*. *davidcooki* were not significant.

## Discussion

To our knowledge, this study is the first investigation of the diet of water mites based on the analysis of DNA in the gut contents of water mites from nature using next-generation sequencing. This work shows that two species of *Lebertia* have highly diverse diets as evidenced by DNA from multiple types of organisms associated with each mite. The method used here, next-generation sequencing analysis, expands on the work that used PCR with 18S primers and lab-fed mites [18] to show the retention of prey DNA by mites after ingestion. The use of next-generation sequencing with mLep/LCOI primers in the present study enabled amplification of non-mite DNA in mites not only from chironomids, which were the sole target of the studies of Martin et al. [18], but also from oligochaetes, other dipterans including mosquitos, and crustaceans. The data provide evidence for seasonal variation in water mite diets. Furthermore, the presence of predation on mosquitoes, combined with the previously well studied parasitic associations with emerging adult mosquitoes indicates that impacts on mosquitoes may be a potentially important ecosystem service provided by water mites.

The hypothesis upon which the present work is based is that non-mite DNA amplified from field-collected water mites represents DNA from organisms that the mite has ingested. Diet studies in other organisms have shown that molecular technologies such as next-generation sequencing can help unravel previously unknown trophic complexities [14]. Studies of spiders (a terrestrial relative of water mites) using molecular sequencing methods revealed prey choices from terrestrial or marine sources [13]. Studies of wild octopus revealed up to 122 molecular taxonomic units (potentially different prey items) that would not have been possible to identify by morphological examination [33].

The results of the next-generation sequencing analysis revealed great prey biodiversity for both *L*. *quinquemaculosa* and *L*. *davidcooki*. Many mites had high proportions and high species identity of DNA barcodes to multiple species of chironomids (see Figs 3 and 4). Recent studies using correlative data on water mite prey complexity demonstrated that copepod, cladoceran and chironomid densities correlated with water mite abundance and species richness [34, 35]. However, this was based on correlation and not on direct observation of water mites feeding or DNA studies, the latter being unique to this present study. As stated in Pozojevic, Jursic [34], molecular studies would increase our understanding of water mite food web functional contributions such as the presence of Oligochaetes in water mite diet which was not reported in the Pozojevic, Jursic [34] study. The results seen here helps us understand some of the ecological significance of the high biodiversity of chironomids revealed in previous studies of the region [36]. For example, our barcode study of chironomids in Maumee Bay and Maumee River (Toledo, OH) revealed up to 45 chironomid operational taxonomic units [36], many of which are similar or identical to those we found with NGS in water mite diets. Our laboratory continues to expand this list of chironomid species in the Great Lakes region (unpublished work), identifying many new barcodes that may be represented in water mite diets.

This work is the first to show evidence of oligochaetes as a significant part of the diet of water mites. We have found only one mention, without supporting evidence, that water mites may feed on oligochaetes [37]. The present study showed highly diverse oligochaete sequences including many whose percent identities to known sequences in GenBank are less than 90% (see Figs 3B and 3C); these sequences could potentially represent new previously unidentified or cryptic species. Erseus et al. [38] have suggested that morphospecies with intraspecific COI barcodes differing by > 9% are likely to comprise cryptic species. We interpret this to mean that we have scarcely scratched the “benthic surface” in understanding the full extent of variation and diversity among oligochaete species in Blue Heron Lagoon and that the study of water mite diets may be an excellent method for discovering oligochaete diversity and unravelling benthic food web trophic dynamics.

The ingestion of chironomids, cladocerans, and oligochaetes in the diet of water mites may be in direct competition with fish, of which many secondary consumers favor these small invertebrates as part of their diets. Previous studies showed that various species of water mites can reduce the standing crop of chironomids by 50% [6] and to equal the amount of predation of cladocerans by fish [9]. By far, the most abundant prey item found in *Lebertia* water mites was chironomids. We also show that *Lebertia* preys on two cladocerans namely *Chydorus brevilabris* (see Fig 6D, preyed by *L*. *quinquemaculosa*) and *Diaphanosoma* (see Fig 6A and 6E, preyed by both *L*. *davidcooki* and *L*. *quinquemaculosa*). Cladoceran *Chydorus brevilabris* is a species complex with high biodiversity that is an important food source for many aquatic organisms [39]. This is just one of several prey items our study has identified which play key roles in food web dynamics. One of the crucial questions that arise from our study. Could *Lebertia* and other water mites be adding to the top down effect on zooplankton prey availability?

This paper also demonstrates seasonal changes in the occurrence of the two *Lebertia* species in Blue Heron Lagoon and differences between the two water mite species in their diets (see Fig 8 and S1 Table). Differences between the diets of *L*. *quinquemaculosa* and *L*. *davidcooki* may reflect seasonal differences in the presence of the two species of *Lebertia* and prey availability. Most specimens of *L*. *quinquemaculosa* (15 out of 20) were collected in October and November 2016, while 20 out of 29 specimens of *L*. *davidcooki* were collected from February to April 2017, and only four of *Lebertia* sp. were collected in October and November 2016. The partial allochrony of these two species may be the mechanism by which these two sympatric species survive in the same geographic area. The quantitative and qualitative differences in both species of *Lebertia* (significant differences in proportions of chironomids and oligochaetes and appearance of ostracod *Podocopida* respectively) may be another mechanism enabling this sympatric relationship. We have previously noted that significant seasonal differences in richness and diversity of water mites in Blue Heron Lagoon may occur as an adaptive response to an intermediate seasonal disturbance [20]; maintenance of sympatry may be another explanation for these seasonal variations. Sympatry may also be maintained by the size difference between the two *Lebertia* species as predator size (see Fig 1) may mediate niche partitioning among various prey choices [20].

The novel observation that adult *Lebertia* prey upon mosquito larvae is supported by both laboratory experiments (Fig 2) and molecular data (Fig 4). The observed mosquito species, *Culex pipiens*, is a known vector of West Nile virus [40]. Both species of *Lebertia* were shown here to ingest *C*. *pipiens*, but as a percentage of the sequences and of all specimens examined, the amount of *C*. *pipiens* in their diets is low (Fig 7). However, Blue Heron Lagoon is less than ideal habitat for mosquitoes, which favor fish-free stagnant waters [41], compared to the lentic habitat but nevertheless wind-affected fishery habitat. An important study, which we have started, is to test the effect of *Lebertia* water mites or other water mite genera found in temporary pools where mosquitoes are generally found [12]. As opportunistic feeders, *Lebertia* in Blue Heron Lagoon may simply not have had many mosquito larvae to choose from compared to chironomid larvae and oligochaetes. Future experiments may apply the techniques demonstrated here to water mites living in more stagnant waters, also found on Belle Isle and known to have a plethora of water mite species (pers. obs.). Given that mosquitoes are vectors of many human diseases (malaria, Zika, West Nile virus, etc.) and therefore among the most harmful insects known, the predatory behavior of water mites, along with the ability of *Lebertia* larvae to parasitize adult mosquitoes [42], indicate that the negative impacts of water mites on mosquitos may be an important ecosystem service provided by water mites [12].

Other ecosystem services provided by water mites include their potential roles in protecting local species from invasive species and their potential uses as bioindicator species. Water mites have been described as useful in containing an invasion of water boatman insects in waters of the Iberian Peninsula [10]. Some species of the Dipteran larvae *Megaselia* (prey item for the mite illustrated in Fig 6E) are known as scuttle flies and their larvae are known to inhabit water filled containers and wet areas in urban habitats [43]. They are of interest due to their ability to infest human corpses [43]. Comparably, previous work has investigated the potential use of water mites as a forensic tool for determining post-mortem timelines of corpses in aquatic habitats [44]. Water mites also represent a potentially important bioindicator of water quality for which studies have been conducted in Central America and Europe [45–47].

A 1989 review by Proctor and Pritchard [5] was the last comprehensive work reviewing the diet of water mites. This seminal work was based on the literature available at the time on water mite diets and included some original observations by the authors as well. However, their review is over 30 years old and an update is due. They stated that beyond laboratory observation, very few “feasible alternatives” were available [5]. With the advent of next-generation sequencing, we have upgraded water mite diet studies and propose to extend these studies beyond the *Lebertia* genus on which the present study focused. Indeed, initial comparisons to *Arrenurus* (e.g., Fig 6) already showed interesting differences and confirmation of ostracods in their diet [5, 34, 48].

## Conclusions

We predict that expanding similar research on other water mite genera will result in a significant body of evidence that would support the importance of water mite predatory behavior in freshwater ecosystems. Their food web importance may have been largely overlooked in past literature and research, viz.: a survey of species-level DNA barcodes for Great Lakes invertebrates in 2015 listed water mites as “nil” in the public databases [49]. Sampling reports on groups in ecological studies often just group water mites in the “other” category or simply “Acari”, diminishing their potential importance at species or even generic level by omission. Since then, we have improved this database, highlighting the great diversity of water mites in several Great Lakes sites [2, 20]. Given the predator-prey interactions that the present work reveals, this update on current knowledge of water mites prey in the Great Lakes region may help gain increased recognition of these organisms in food web dynamics and a greater appreciation of their ecosystem services.

## Supporting information

S1 Table Water mite specimens with dates of collection and gut contents. (PDF)

S2 Table Non-mite DNA in water mites from nature. (PDF)
