# Supplementary material for: Molecular diet studies of water mites reveal prey biodiversity
Source: PLoS One. 2021 Jul 29;16(7):e0254598. doi: 10.1371/journal.pone.0254598 (PMC8321515; doi:10.1371/journal.pone.0254598)
Supplement: S1 Table — (PDF) [file pone.0254598.s001.pdf]

S1 Table. Water mite specimens with dates of collection and gut contents.

S1 Table. Water Mite Specimens: Dates of Collection and Gut Contents

Specimen ID	Mite species	Date collected	Percent of Gut Sequences Classified As					
			Chironomidae	Oligochaeta	Mosquito	Ostracoda	Cladocera	Other
BHL040916-Ldc69	*L. davidcooki*	4/9/2016	98.82%	0.00%	0.00%	0.00%	0.16%	1.02%
BHL072216-Lq68	*L. quinquemaculosa*	7/22/2016	47.49%	0.00%	0.00%	0.00%	46.31%	6.20%
BHL072216-Arr95	*Arrenurus sp.*	7/22/2016	3.96%	0.00%	0.00%	95.23%	0.00%	0.81%
BHL072216-Arr96	*Arrenurus sp.*	7/22/2016	85.30%	0.00%	0.00%	0.35%	0.00%	14.35%
BHL101416-Lq60	*L. quinquemaculosa*	10/14/2016	62.80%	27.60%	2.40%	0.00%	7.20%	0.00%
BHL101516-Ldc85	*L. davidcooki*	10/15/2016	80.51%	0.00%	0.00%	19.49%	0.00%	0.00%
BHL101516-Ldc86	*L. davidcooki*	10/15/2016	89.19%	0.00%	0.00%	9.38%	0.51%	0.92%
BHL101516-Lq78	*L. quinquemaculosa*	10/15/2016	0.00%	98.26%	0.00%	0.00%	0.00%	1.74%
BHL101516-Lq79	*L. quinquemaculosa*	10/15/2016	1.28%	95.55%	0.00%	0.00%	0.00%	3.17%
BHL101516-Lq80	*L. quinquemaculosa*	10/15/2016	96.87%	2.74%	0.00%	0.00%	0.00%	0.39%
BHL101516-Lq81	*L. quinquemaculosa*	10/15/2016	0.00%	100.00%	0.00%	0.00%	0.00%	0.00%
BHL101516-Lq82	*L. quinquemaculosa*	10/15/2016	30.41%	64.33%	0.00%	0.00%	0.00%	5.26%
BHL101516-Lq83	*L. quinquemaculosa*	10/15/2016	65.63%	30.63%	0.00%	0.00%	0.00%	3.74%
BHL101516-Lim84	*Limnesia sp.*	10/15/2016	0.11%	98.08%	0.00%	0.00%	0.00%	1.80%
BHL110116-Ldc73	*L. davidcooki*	11/1/2016	46.09%	53.91%	0.00%	0.00%	0.00%	0.00%
BHL110116-Ldc71	*L. davidcooki*	11/1/2016	91.85%	0.00%	0.00%	0.00%	7.32%	0.83%
BHL110116-Lq44	*L. quinquemaculosa*	11/1/2016	70.69%	26.64%	0.00%	0.00%	0.00%	2.67%
BHL110116-Lq45	*L. quinquemaculosa*	11/1/2016	46.49%	51.30%	0.00%	0.00%	0.00%	2.21%
BHL110116-Lq46	*L. quinquemaculosa*	11/1/2016	31.15%	66.69%	0.00%	0.00%	0.00%	2.17%
BHL110116-Lq47	*L. quinquemaculosa*	11/1/2016	25.82%	69.64%	0.00%	0.00%	0.00%	4.54%
BHL110116-Lq74	*L. quinquemaculosa*	11/1/2016	38.97%	61.03%	0.00%	0.00%	0.00%	0.00%
BHL110116-Lq75	*L. quinquemaculosa*	11/1/2016	48.22%	51.78%	0.00%	0.00%	0.00%	0.00%
BHL110116-Lq76	*L. quinquemaculosa*	11/1/2016	37.97%	62.03%	0.00%	0.00%	0.00%	0.00%
BHL110116-Lq77	*L. quinquemaculosa*	11/1/2016	54.37%	43.67%	0.00%	0.00%	0.00%	1.95%
BHL022317-Ldc87	*L. davidcooki*	2/23/2017	82.87%	0.00%	0.00%	15.58%	0.00%	1.56%
BHL022317-Ldc88	*L. davidcooki*	2/23/2017	69.61%	0.00%	0.00%	30.39%	0.00%	0.00%
BHL022317-Ldc89	*L. davidcooki*	2/23/2017	83.69%	0.00%	0.00%	14.62%	0.00%	1.69%
BHL022317-Ldc90	*L. davidcooki*	2/23/2017	74.86%	0.00%	0.00%	25.14%	0.00%	0.00%
BHL022317-Ldc91	*L. davidcooki*	2/23/2017	70.77%	2.96%	0.00%	0.14%	0.00%	26.13%
BHL022317-Ldc92	*L. davidcooki*	2/23/2017	98.18%	0.19%	0.00%	0.00%	0.00%	1.64%
BHL032417-Ldc25	*L. davidcooki*	3/24/2017	63.78%	27.32%	0.00%	0.00%	0.00%	8.90%
BHL032417-Ldc26	*L. davidcooki*	3/24/2017	100.00%	0.00%	0.00%	0.00%	0.00%	0.00%
BHL032417-Ldc28	*L. davidcooki*	3/24/2017	89.11%	0.80%	7.49%	0.00%	0.00%	2.60%
BHL032417-Ldc29	*L. davidcooki*	3/24/2017	92.02%	0.00%	0.00%	0.00%	0.00%	7.98%
BHL032417-Ldc30	*L. davidcooki*	3/24/2017	93.76%	0.04%	0.00%	0.00%	0.00%	6.24%
BHL032417-Ldc31	*L. davidcooki*	3/24/2017	95.31%	0.06%	0.00%	0.00%	0.00%	4.69%
BHL032417-Ldc32	*L. davidcooki*	3/24/2017	87.49%	0.03%	9.85%	0.00%	0.00%	2.65%
BHL032417-Ldc33	*L. davidcooki*	3/24/2017	93.36%	2.30%	0.00%	0.00%	0.00%	4.35%
BHL032417-Ldc34	*L. davidcooki*	3/24/2017	57.18%	37.45%	0.00%	0.00%	0.00%	5.37%
BHL032417-Lq48	*L. quinquemaculosa*	3/24/2017	95.50%	0.00%	0.32%	0.00%	0.00%	4.18%
BHL032417-Lq56	*L. quinquemaculosa*	3/24/2017	23.17%	0.10%	0.00%	0.00%	74.58%	2.15%
BHL040517-Ldc35	*L. davidcooki*	4/5/2017	68.15%	23.91%	0.00%	0.00%	3.28%	4.67%
BHL040517-Ldc36	*L. davidcooki*	4/5/2017	100.00%	0.04%	0.00%	0.00%	0.00%	0.00%
BHL040517-Ldc61	*L. davidcooki*	4/5/2017	88.85%	7.16%	0.00%	0.00%	0.00%	4.00%

BHL040517-Ldc62	*L. davidcooki*	4/5/2017	97.31%	0.00%	0.00%	0.00%	0.00%	2.69%
BHL040517-Ldc63	*L. davidcooki*	4/5/2017	100.00%	0.00%	0.00%	0.00%	0.00%	0.00%
BHL040517-Ldc64	*L. davidcooki*	4/5/2017	100.00%	0.00%	0.00%	0.00%	0.00%	0.00%
BHL040517-Ldc65	*L. davidcooki*	4/5/2017	94.53%	0.00%	0.00%	0.00%	0.00%	5.47%
BHL040517-Ldc66	*L. davidcooki*	4/5/2017	85.22%	0.12%	0.00%	0.00%	0.00%	14.66%
BHL040517-Ldc67	*L. davidcooki*	4/5/2017	100.00%	0.00%	0.00%	0.00%	0.00%	0.00%
BHL040517-Lq57	*L. quinquemaculosa*	4/5/2017	74.84%	0.00%	21.33%	0.00%	0.00%	3.83%
BHL040517-Lq58	*L. quinquemaculosa*	4/5/2017	58.18%	33.81%	2.55%	0.00%	0.00%	5.46%
