# Supplementary material for: Molecular diet studies of water mites reveal prey biodiversity
Source: PLoS One. 2021 Jul 29;16(7):e0254598. doi: 10.1371/journal.pone.0254598 (PMC8321515; doi:10.1371/journal.pone.0254598)
Supplement: S2 Table — (PDF) [file pone.0254598.s002.pdf]

S2 Table. Non-mite DNA in water mites from nature.

**Water mite species (sample ID)**	**GenBank match***
Lebertia davidcooki (8-BHL022317)	*Chironomidae* sp. Q95%; ID87% KP045212.1
Lebertia davidcooki (6-BHL022317)	*Paratanytarsus* sp. Q93%; ID100% KM988017.1
Lebertia sp. (2-BHL022317)	*Paratanytarsus* sp. Q75%; ID99% KR276527.1
L. quinquemaculosa (138-BHL110116)	*Slavina appendiculata* Q85%; ID 88% GQ355375.1
L. quinquemaculosa (145-BHL110116)	*Nais elinquis* Q88%; ID87% GQ355369.1
L. quinquemaculosa (8-BHL072216)	*Diaphanosoma* sp. Q42%; ID99% LC060041.1

*Closest match in GenBank of sequences of PCR products obtained with mLep/LCO1490 primers. Listed as *taxon* of closest match, query coverage (Q), % identification (ID), GenBank accession ID
